# Curcumin in Metabolic Health and Disease

**DOI:** 10.3390/nu13124440

**Published:** 2021-12-11

**Authors:** Marzena Jabczyk, Justyna Nowak, Bartosz Hudzik, Barbara Zubelewicz-Szkodzińska

**Affiliations:** 1 Department of Nutrition-Related Disease Prevention, Faculty of Health Sciences in Bytom, Medical University of Silesia, Piekarska 18 Street, 41-902 Bytom, Poland; marzena.jabczyk@gmail.com (M.J.); bzubelewicz-szkodzinska@sum.edu.pl (B.Z.-S.); 2 Department of Cardiovascular Disease Prevention, Faculty of Health Sciences in Bytom, Medical University of Silesia, Piekarska 18 Street, 41-902 Bytom, Poland; bartekh@mp.pl; 3 Silesian Center for Heart Diseases, Third Department of Cardiology, Faculty of Medical Science in Zabrze, Medical University of Silesia, 41-800 Zabrze, Poland

**Keywords:** curcumin, nanocurcumin, metabolic diseases, polycystic ovary syndrome, metabolic syndrome, non-alcoholic fatty liver syndrome, atherosclerosis

## Abstract

In recent years, epidemiological studies have suggested that metabolic disorders are nutritionally dependent. A healthy diet that is rich in polyphenols may be beneficial in the treatment of metabolic diseases such as polycystic ovary syndrome, metabolic syndrome, non-alcoholic fatty liver disease, cardiovascular disease, and, in particular, atherosclerosis. Curcumin is a polyphenol found in turmeric and has been reported to have antioxidant, anti-inflammatory, hepatoprotective, anti-atherosclerotic, and antidiabetic properties, among others. This review summarizes the influence of supplementation with curcumin on metabolic parameters in selected metabolic disorders.

## 1. Introduction

Curcumin (1,7-bis(4-hydroxy-3-methoxy phenol)-1,6-heptadiene-3,5-dione) is a lipophilic polyphenol classified as a curcuminoid. It has, among others, antioxidant, anti-inflammatory, and anticancer effects [1,2]. Indeed, today, its use is being explored in the course of many diseases; however, the history of the use of curcumin dates back several thousand years [3] due to its culinary use and health-promoting properties. The source of curcumin is turmeric (*Curcuma longa*), a plant from the ginger family that grows in Asia, particularly in India. There, turmeric is often used as a spice due to its intense yellow color, aroma, and flavor [1]. Unfortunately, the curcumin content in turmeric is only a few percent, and it has very low bioavailability [3]. For this reason, the use of curcumin for therapeutic purposes occurs through standardized supplements, which have an increased bioavailability of curcumin by up to 2000% [3]. Hence, there is another limitation in establishing a proper therapeutic dosage of curcumin. Curcumin metabolism may be different from person to person due to the variety of microbiota [4]. One of the methods of enhancing the biological and pharmacological activity of curcumin is nanoencapsulation. Over recent years, many formulations of curcumin have been developed in order to increase its bioavailability and solubility and to shield it from inactivation through hydrolysis [5,6,7,8,9,10,11,12,13].

The most beneficial nanoformulations of curcumin are with liposomes, polymers, gold nanoparticles, magnetic nanoparticles, solid lipid nanoparticles (SLNs), conjugates, cyclodextrins, solid dispersions, micelles, nanospheres, nanogels, and nanodisks. Each of these confirms its usefulness in the diagnosis of various diseases due to the very different outcomes. For example, liposomes have the ability to control the release of drugs with an optimal molecular ratio; they reduce side effects and toxicity in cells, and despite their short half-life, are rapidly removed by the reticuloendothelial system or due to their poor stability [6]. In turn, polymers (polymeric nanoparticles) are safe, effective, and biocompatible due to their small size, and thus have the ability to circulate in the blood for a long time [5,7]. Gold nanoparticles are useful in prostate and colorectal cancer cell models [8] due to their optical, catalytic, and non-toxic properties [5]. Magnetic nanoparticles also have some advantages. Their low production cost and the sustainable delivery of thiolated starch-coated iron oxide may indicate the compatibility of this system with lymphocyte cells. Additionally, magnetic nanoparticles may by manipulated in terms of their forms, shapes, sizes, and chemical properties [9]. To date, magnetic nanoparticles with curcumin (MNP@PEG-Cur) have been established as a recommendation for drug carriers for antitumor medicines due to their high biocompatibility [10]. SLNs may also be useful for cancer treatment [5]. It has been shown that they enhance the solubility compared to that of native curcumin and have the ability to reduce the activity of lipopolysaccharides (LPSs) [11]. Two or more molecules may be formed into conjugates [5]. Conjugation of curcumin with molecules and hydrophilic polymers is efficient due to the increase in solubility and oral bioavailability. This formulation may be a potential treatment for Alzheimer’s disease [12]. Cyclodextrins are used to enhance drug stability and solubility and to deliver drugs to cancer cells in their active form. These forms have the ability to bear non-covalently bound drugs [5]. Other forms such as solid dispersions may decrease the particle size to the nano range with improved wettability, pharmacokinetic properties, and oral biodistribution of the drugs [5]. Micelles are widely used with curcumin to deliver poorly water-soluble drugs [13]. Curcumin formulations with nanospheres may present better cytotoxic effects against cancer cells and improve the solubility and stability in comparison to native curcumin, and they may also have great potential as a treatment for Alzheimer’s disease [5]. Various other nanoformulations (nanogels, nanodisks) enhance curcumin’s biological activity. These methods may be beneficial in controlling drug release and circulation and improving anticancer activity [5]. 

**Figure 1:**
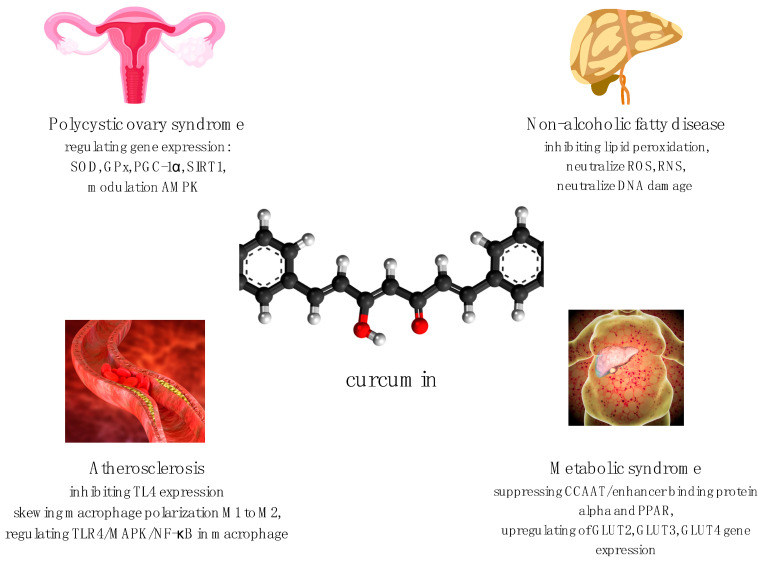
Partial mechanisms of curcumin’s regulation of metabolic properties [15,16,17,18,19,20,21]. SOD—superoxide dismutase; GPx—glutathione enzyme peroxidase; PGC-1 α—peroxisome proliferator-activated receptor γ coactivator-1α; SIRT1—silent information regulator 1; AMPK—5’AMP-activated protein kinase; TL4—toll-like receptor 4; M1—M1 macrophage; M2—M2 macrophage; TLR4/MAPK/NF-κB—toll-like receptor 4/mitogen-activated protein kinase/nuclear factor kappa light chain enhancer of activated B cell pathway; ROS—reactive oxygen species; RNS—reactive nitrogen species; PPAR—peroxisome proliferator-activated receptor; GLUT2—glucose transporter 2; GLUT3—glucose transporter 3; GLUT4—glucose transporter 4.

So far, nanocurcumin has been confirmed to have better bioavailability, chelating properties, and retention time in comparison to curcumin [5], and also seems to have the best effects on gut microbiota [4]. However, this conclusion has been assessed in a limited number of clinical trials [5]. Curcumin has exhibited promising capabilities in both preclinical and clinical studies. Curcumin is now in human trials for various diseases such as polycystic ovary syndrome (PCOS), metabolic syndrome, non-alcoholic fatty liver disease, atherosclerosis, and many more including neurodegenerative conditions [14]. Partial mechanisms of curcumin’s regulation of metabolic properties are shown in Figure 1 [15,16,17,18,19,20,21]. The aim of this paper is to provide an overview of the role of curcumin in some metabolic diseases such as polycystic ovary syndrome, metabolic syndrome, non-alcoholic fatty liver disease, cardiovascular disease, and atherosclerosis. The data from recent years were reviewed, but most of the studies discussed here were conducted within the last two years. In September 2021, on Clinicaltrials.gov, we found 270 records that included curcumin, five studies that included the keyword “nanocurcumin”, and three records with “nano-curcumin”.

### 1.1. Curcumin for Treatment of Polycystic Ovary Syndrome (PCOS)

Polycystic ovary syndrome is one of the major metabolic diseases in women worldwide, particularly in developing countries. This endocrine disorder is associated with insulin resistance, obesity, hypertension, and dyslipidemia as well as with non-metabolic disorders such as cardiovascular diseases and disorders in the reproductive organs, which may occur in the form of infertility, hyperandrogenism, and hirsutism [22]. According to the Androgen Excess and PCOS Society, the following are used to diagnose PCOS: the presence of clinical and/or biochemical hyperandrogenism, ovarian dysfunction (menstrual abnormalities), and the presence of the polycystic ovary morphology [23]. Additionally, PCOS is characterized by intrinsic insulin resistance, which may lead to the occurrence of metabolic syndrome and, as a consequence, to the development of type 2 diabetes mellitus (T2DM). Disturbed carbohydrate metabolism in PCOS patients may occur through insulin-dependent mechanisms [24]. The increase in plasma glucose stimulates insulin secretion via pancreatic beta cells. This may result in the suppression of lipolysis and a decrease in the rate of lipid oxidation. Simultaneously, glucose is stimulated by insulin for uptake in skeletal muscle, thus increasing the glucose outflow [24]. The latest systematic review and meta-analysis of randomized-control trials investigated a significant improvement in fasting glucose, fasting insulin, the homeostasis model assessment measuring insulin resistance (HOMA-IR), and the quantitative insulin sensitivity check index (QUICKI) in women with PCOS who took curcumin in comparison with a placebo group. Improvements in high-density lipoprotein (HDL) and total cholesterol (TC) have also been reported in patients taking curcumin supplements. In the control group, there were no significant differences in low-density lipoprotein (LDL) and triglyceride (TG) levels between curcumin-taking patients and those taking a placebo [25]. Ultimately, the meta-analysis included only three studies, which are discussed later in this paper. The results obtained from the meta-analysis of Chien et al. are summarized in Table 1. 

Jamilian et al. [26] found that administration of curcumin for 12 weeks in women with PCOS had beneficial effects on glycemic control, among other things. The researchers reported that reduced fasting glucose (*p* = 0.002) significantly increased insulin sensitivity (*p* = 0.02), and positive alterations in serum lipids (i.e., a decrease in total cholesterol (*p* = 0.001) and LDL cholesterol (*p* = 0.001) and an increase in HDL cholesterol levels (*p* = 0.01)) in comparison to patients taking a placebo; in addition, curcumin supplements decreased the weight of women suffering from PCOS. Other researchers who have looked at the effects of curcumin on glycemic status, lipid profile, and high-sensitivity C-reactive protein (hs-CRP) levels in overweight/obese women with PCOS found that serum insulin, QUICKI (*p* < 0.05), and HOMA-IR (*p* = 0.067) were significantly improved in the group treated with curcumin. In contrast, the differences in lipid parameters and hs-CRP levels were not statistically significant in the curcumin-treated group [27].

Curcumin may stimulate insulin-mediated glucose uptake through the phosphatidylinositol 3-kinsase (PI3K)/Akt pathway, which, in turn, upregulates glucose transporter 4 (GLUT4) in the adipocyte and skeletal muscle, leading to an increase in glucose levels. Additionally, curcumin may also enhance GLUT4 and glucose uptake in adipocytes [25]. Curcumin has been shown to inhibit liver gluconeogenesis through modulation of 5’AMP-activated protein kinase (AMPK), thus reducing blood glucose levels [15]. Moreover, the lipid-lowering potential of curcumin may be a consequence of curcumin’s ability to decrease the circulatory levels of lipid peroxides and total serum cholesterol (TC), or to increase the levels of high-density lipoprotein (HDL). There are some potential mechanisms that may be responsible for the beneficial influence of curcumin on lipid profile; for example, curcumin may suppress the expression of Niemen-Pick C1-like (NPC1) protein in the intestine, which mediates the cholesterol absorption of hepatocytes [19]. Curcumin also ameliorates dyslipidemia and activates the lipid metabolism pathway, which elevates lipoprotein lipase activity to decrease triglyceride levels [26]. The hypothesis about the beneficial impact of curcumin supplementation on women with PCOS is because curcumin may support the improvement in complications of PCOS by regulating gene expression—that is, by increasing the gene expression of superoxide dismutase (SOD) and glutathione peroxidase enzymes (GPx)—and cellular signaling [15].

However, one of the first studies on the effects of curcumin on postprandial glucose and insulin response, which was conducted in 2010 by Wickenberg et al. [29], only showed the possible effects of curcumin on postprandial insulin levels (after 30 min, *p* = 0.003; after 60 min, *p* = 0.041; after OGTT including *C. longa*). There were no statistically significant differences in the response to glucose. However, the main weakness of the study was the small sample of patients (the group of respondents consisted of only 14 patients; half of them were in the placebo group) as well as the dose (6 g of *C. longa*); moreover, the form of curcumin used, according to the current state of knowledge, is a form of curcumin with poor bioavailability. Tablets containing either a placebo or *C. longa* were administered together with a 75 g oral glucose tolerance test.

Insulin resistance is one of the main mechanisms of PCOS. Curcumin is generally assumed to improve the body’s antioxidant enzymes by impacting related gene expression in patients with PCOS. In a randomized and double-blinded clinical trial involving 67 overweight or obese female patients with PCOS, the effects of curcumin on gene expression of peroxisome proliferator-activated receptor γ coactivator-1α (PGC-1α) (*p* = 0.011) and silent information regulator 1 (SIRT1) were reported [15]. SIRT1 contributes to the deacetylation of the PGC-1α gene, thereby increasing the rate of thermogenesis and oxidation of lipids, and is also an NAD+-dependent histone deacetylase in the pathway of insulin secretion [15]. These results would seem to suggest that curcumin may improve hormonal profiles in patients with PCOS due to its support of ovarian function by reducing inflammation and oxidative stress.

Interestingly, Sohrevardi et al. [30] reported that curcumin has a synergistic effect with metformin in the improvement of insulin resistance and lipid profiles in patients with PCOS. The researchers evaluated the effects of metformin with and without curcumin nano-micelles in the treatment of women with PCOS. Their study showed that the hormonal parameter of total testosterone levels and the biochemical parameters of triglycerides, HDL, and total cholesterol were significantly improved in the group taking curcumin together with metformin after three months in comparison to the group treated with only metformin. HOMA-IR and the insulin sensitivity check index (QUICKI) were also assessed.

The anti-inflammatory properties of curcumin may mitigate hyperandrogenism due to its possible role in glucose and lipid metabolism. Moreover, curcumin has the ability to lower circulating tumor necrosis factor alpha (TNF-α) and interleukin 6 (IL-6) concentration. Proinflammatory cytokines such as TNF-α have been found to be significantly higher in PCOS patients [25]. Moreover, curcumin may exert anti-diabetic effects by increasing the gene expression of PPAR-γ, which has a pleiotropic impact on glucose homeostasis and insulin sensitivity and controls gene expression in lipid and glucose metabolism [26]. Heshmati et al. [28] reported not only reduced glucose and insulin plasma levels, but also significantly reduced serum dehydroepiandrosterone (DHEA) levels (−26.53 μg/dL; *p* = 0.035) in patients with PCOS who took curcumin supplements for 12 weeks in doses of 1500 mg per day in comparison to the placebo patients. To date, there are few studies that have assessed the influence of curcumin on the sex hormones in women with PCOS.

Regarding proinflammatory cytokines, Mohammadi et al. [31] investigated the therapeutic effects of curcumin on TNF-α, IL-6, and C-reactive protein (CRP) in rats with PCOS. The difference between the curcumin-treated group and the non-curcumin-treated rats with PCOS was significant. The results showed decreased IL-6 and CRP, and interestingly, they observed decreased expression of tumor necrosis factor alpha (TNF-α) in the granulosa layer and follicular fluid of follicles and ovarian cysts in the PCOS group treated with curcumin. In a recent study by Abuelezz et al. [32], it was found that nanocurcumin significantly improved oxidative markers, glucose indices, insulin resistance, and TNF-α levels in a PCOS-induced animal model. The CRP and IL-6 levels were also decreased in other studies [33].

Turning to the experimental evidence on a mouse model of PCOS, Abhari et al. [34] found that ovarian injures and DHEA-induced apoptosis were efficiently suppressed by curcumin. These results suggest that curcumin may indicate the protective properties of nanoparticles of curcumin against PCOS.

Most studies of curcumin and PCOS were only carried out among a small number of respondents. Therefore, there is still a need for larger randomized clinical trials to determine the possible beneficial effects of curcumin on PCOS.

### 1.2. Curcumin for Treatment of Metabolic Syndrome

Metabolic syndrome (MS) is a highly prevalent and complex metabolic disorder that includes central obesity, dyslipidemia, elevated blood glucose levels, hypertension, and insulin resistance [35]. MS is currently estimated to affect approximately 20% of the global population. In diagnosing MS, there are some variations in the metabolic components thought to comprise it depending on (e.g., sex or ethnicity). Overall, the following are commonly recognized: impaired glucose tolerance or diabetes, obesity, hypertension, and/or dyslipidemia [36]. Curcumin has been shown to have a role in decreasing angiogenesis and adipogenesis by suppressing CCAAT/enhancer-binding protein alpha and PPAR expression and by lowering cholesterol levels. Moreover, curcumin has the ability to upregulate the gene expression of pancreatic glucose transporter 2 (GLUT2), GLUT3, and GLUT4, thus stimulating insulin secretion [16]. In a randomized and double-blinded clinical trial, Bateni et al. [37] investigated the effects of curcumin nano-micelles in patients with metabolic syndrome. The study demonstrated that supplementation with 80 mg/day of curcumin nano-micelles for 12 weeks significantly reduced the plasma TG levels in comparison to those of the placebo group (*p* = 0.03) as well as the homeostasis model assessment of ß-cell dysfunction (HOMA-ß). However, there were no beneficial effects on insulin, HOMA-IR, glycosylated hemoglobin (HbA1c), or fasting blood sugar (FBS). Moreover, no changes in anthropometric indices or blood pressure were reported. These results must be approached with some caution because of the limitations of the study. First of all, the inability to measure plasma levels of curcumin in patients, which can be different due to the large range of body mass indices (BMIs), may have been a factor in the different responses to curcumin. Second, the long-term effects of consumption of nano-curcumin formulations remain unknown. Furthermore, it is crucial to pay attention to the dose of nano-curcumin supplements. In another study, it was observed that using 200 mg/kg of nano-curcumin had a greater therapeutic effect in diabetic rat samples in comparison to using 100 mg/kg of nano-curcumin [38].

The interesting results presented by Kheiripour et al. [35] suggest that supplementation with nano-curcumin in doses of 80 mg/per day for two months may be beneficial in patients diagnosed with MS. They reported that after treatment with nano-curcumin, the values of FPG, insulin, HOMA-IR, and afamin were decreased (*p* = 0.047). Afamin is a glycoprotein, and has been shown to have a relation to obesity, PCOS, type 2 diabetes, high blood pressure, and dyslipidemia. Moreover, further analysis showed that there was also a significant negative correlation between afamin and HDL levels (*p* = 0.044). 

The effects of nano-curcumin therapy on metabolic parameters in two clinical trials and in one study of patients with metabolic syndrome and type 2 diabetes mellitus are presented in Table 2. The statistically significant results are highlighted.

Diabetes mellitus is defined as a metabolic disease related to hyperglycemia, and it results from insulin resistance or problems with insulin action or insulin secretion. The influence of nano-curcumin on type 2 diabetic patients was the foundation of the study by Rahimi et al. [39]. The researchers investigated the effects of nano-curcumin on parameters such as HbA1C, fat, and blood glucose, and lipid profiles in a diabetic group. The results of the three-month double-blind randomized clinical trial with a dosage of 80 mg/day of curcumin in the form of nano-micelles confirmed that there were significant differences in HbA1c and LDL-C levels and BMI, but there were no statistical differences in fasting glucose levels, TC, HDL-C levels, or TG levels. 

Now, the EPICURO study has begun, which aims to demonstrate the beneficial effects of a 6-month dietary supplement of turmeric with an improved bioavailability (MERIVA^®^) on inflammatory, oxidative, and metabolic parameters as well as cognitive performance in patients with metabolic syndrome (NCT04705220).

### 1.3. Curcumin for Non-Alcoholic Fatty Liver Disease (NAFLD)

Non-alcoholic fatty liver disease (NAFLD)—the nomenclature of which was recently updated to metabolic-associated fatty liver disease (MAFLD)—is the most common chronic liver disease worldwide [40]. It is characterized by excessive fat accumulation that is greater than 5% of the total weight of the liver. The development of liver disease—and, in particular, NAFLD—involves oxidative stress processes and damage to lipids, proteins, and DNA as well as changes in functional signaling pathways [18]. Curcumin and related phenolics have been linked with the inhibition of lipid peroxidation, free radical formation (e.g., neutralization of superoxide, peroxyl, and hydroxyl radicals (ROSs), nitric oxide, and peroxynitrite (RNS)) and DNA damage. Despite obesity [17] and hyperlipidemia [18], it is also known that patients with type 2 diabetes have a high prevalence of NAFLD (up to 70%). The above diseases share multiple cardiometabolic risk factors and proinflammatory pathways [18]. Różański et al. [1] analyzed databases and publications that have described the effects of using curcumin supplementation on biochemical parameters in MAFLD. They concluded that curcumin may have therapeutic potential in MAFLD patients; however, the analyzed studies did not allow them to clearly determine its positive effects. 

In a similar case, Jalali et al. [41] included nine relevant randomized controlled trials (RCTs) in their meta-analysis in order to study the effects of curcumin supplements on metabolic markers and anthropometric parameters in patients with NAFLD. As shown in Table 3, the study reported a significant decrease not only in alanine transaminase (ALT) (*p* = 0.049) and aspartate transaminase (AST) (*p* = 0.032) activity, but also in serum total cholesterol (TC), LDL, FBS (*p* = 0.027), HOMA-IR (*p* = 0.031), serum insulin, and waist circumference (WC). No significant reductions in the biochemical parameters of TG, HDL, HbA1c, body weight, or, therefore, BMI following curcumin supplementation were observed. However, after a meta-regression analysis of the duration and a dosage-based analysis, a significant change in BMI was indicated, and a subgroup analysis (age-based and TC-based) also indicated a significant decrease in TG. The study investigated changes in two-month and three-month supplementation with curcumin. The authors concluded that the use of curcumin in the analyzed studies had a beneficial effect on both metabolic and anthropometric parameters in patients with NAFLD. Ultimately, more research is needed with larger groups of patients and adequate durations in order to establish efficient curcumin therapies to improve liver function.

Currently, there is a study underway (in the recruitment phase) at the Columbia University Medical Center to evaluate the effects of curcumin supplementation on NAFLD in pediatric patients (NCT04109742).

### 1.4. Curcumin for Treatment of Atherosclerosis

Inflammatory responses, oxidative stress, lipid deregulation, and epigenetic disorders may derive from chronic problems in arteries, defined as atherosclerosis [19,20,42]. Curcumin limits the risk of lipid peroxidation, which triggers inflammatory responses that may lead to cardiovascular disease (CVD) and atherosclerosis, due to its ability to scavenge reactive oxygen forms. Moreover, curcumin and statins influence the same mediators of plasma lipid changes [19]. Ashtary-Larky et al. [33], in their meta-analysis of clinical trials, determined risk factors for cardiovascular disease after nano-curcumin supplementation. The meta-analysis showed that nano-curcumin may demonstrate anti-inflammatory effects; thus, it decreases CRP and IL-6 levels (*p* < 0.001). According to the results of the study, there were improvements in the glycemic profile as a result of lowering the fasting blood glucose (*p* < 0.001) and increasing the HDL (*p* < 0.001) due to nano-curcumin supplementation. Interestingly, the levels of other lipid profile markers—LDL, TC, and TG—were subject to more beneficial effects in patients with dyslipidemia at baseline. Table 4 presents the results obtained from the meta-analysis of nano-curcumin with respect to the metabolic parameters. The statistically significant results are highlighted.

Turning now to experimental studies on atherosclerosis, in their meta-analysis, Lin et al. [43] concluded that the positive effects of curcumin on atherosclerosis were associated with the dose of curcumin, and a medium dosage of curcumin (100–200 mg/kg body weight) was confirmed to have better results than the predicted dosage, which was above 200 mg/kg body weight per day. Additionally, curcumin has the ability to prevent endothelial dysfunction and smooth muscle cell proliferation and migration. It also modulates macrophage polarization and counteracts inflammatory response, which may be beneficial in the treatment of atherosclerosis [20]. By scavenging and presenting antigens, monocyte-derived macrophages contribute to the immune and inflammatory response and, as a consequence, play a role in the initiation and progression of atherosclerosis [21,44]. These properties of curcumin are responsible for skewing macrophage polarization from M1 to M2, regulating TLR4/MAPK/NF-κB pathways in macrophages (which induce M2 polarization) and secreting interleukins (IL-4 and/or IL-13). Moreover, curcumin may indirectly maintain cell homeostasis by regulating the expression and activity of lipid transporter, which is responsible for cholesterol uptake and efflux [20]. Zhou et al. [21] suggested that curcumin could be used as a therapeutic supplement in atherosclerosis due to its ability to modulate macrophage polarization through the inhibition of the toll-like receptor (TL4)-mediated signaling pathway. This indicates that curcumin is related to anti-inflammatory and atheroprotective effects. TL4 is a signaling receptor that plays a key role in the pathogenesis of plaque formation and the development of atherosclerosis. Activation of TL4 leads to the activation of NF-κB and MPK, and thus triggers nuclear transduction, which simultaneously propels the gene expression profile of an inflammatory response. This is a cause of the increased ROS production and the expression of inflammation [20,45]. Zhang et al. [45] investigated the potential suppression of atherosclerosis development by curcumin in ApoE-knockout mice by inhibiting TLR4 expression in an animal model. Mice were fed a high-fat diet supplemented with curcumin for 16 weeks and compared to a control group (without curcumin supplementation). The results indicated that, in vitro, curcumin at least partially inhibited TLR4 expression, inhibited NF-κB activation in macrophages, and, indeed, influenced the inflammatory reaction. The causal role of curcumin in inhibiting TLR4 expression was also demonstrated by Meng et al. [46], who indicated that its mechanism may be related to the blocking of NADPH-mediated intracellular ROS production.

Comprehensively, the treatment of atherosclerosis and other cardiovascular diseases with curcumin was shown to be effective in many studies. Given the positive potential of curcumin supplementation, it is necessary to carry out many other high-quality studies and to gain clinical evidence based on randomized clinical trials on populations with atherosclerosis. This would be helpful in establishing dietary supplementation in a reliable therapeutic schedule [43]. 

## 2. Summary

Curcumin supplementation in metabolic disorders has been the subject of many clinical studies in recent years. The results presented in this review suggest its beneficial effects on metabolic parameters in some metabolic diseases such as polycystic ovary syndrome, metabolic syndrome, glycemic disorders, non-alcoholic fatty liver disease, and cardiovascular disease—particularly atherosclerosis. Mostly, improvements have been observed in fasting glucose, fasting insulin, HOMA-IR, and lipid profiles, which may be the result of curcumin’s ability to mediate many signaling pathways, decrease inflammatory response, or indirectly maintain cell homeostasis. Recent studies have found that the effects of nano-curcumin on serum lipid profiles are more effective than those of curcumin. A possible explanation for these results may be the increased solubility and bioavailability of nano-curcumin, which are probably dose-dependent. However, the limitations of curcumin supplementation prevent the establishment of therapeutic dosages of curcumin in the treatment of metabolic diseases due to the poor bioavailability and rapid metabolism of curcumin.

Several limitations of the present studies should be mentioned. First, most of the results included had a small number of participants, and the number of studies was limited. Second, all studies lasted three months or less; as such, it is not possible to present the long-term effects of curcumin and nano-curcumin supplementation on metabolic diseases and overall health. Third, a relatively high number of the studies included were conducted in Iran, and there was notable heterogeneity among the studies. Fourth, various dosages and sources of curcumin and nano-curcumin were used in the included studies. Among the studies, the curcumin dosages ranged from 40 mg of nano-curcumin to 1500 mg of curcumin per day, and the treatment periods ranged from two to three months.

There is a problem with establishing doses of formulations that would induce side effects due to the lack of studies of the long-term effects of curcumin supplementation. Researchers who analyzed the curcumin supplementation in PCOS patients concluded that curcumin may improve glycemic and lipid metabolism in patients with PCOS without adverse effects. 

The studies analyzed here concluded that curcumin supplementation mostly has advantages (all studies presented several significant improvements in the study area), while the disadvantages were omitted or relatively limited, which may be the consequence of the short-time periods of these studies in the area of curcumin supplementation, especially for metabolic disorders.

In conclusion, it is recommended that the most available form of curcumin (e.g., nano-curcumin) be used to obtain the best therapeutic results, especially with metabolic and hormonal diseases. Another possible area of future research would be to investigate the most optimal doses of curcumin supplements in order to establish the ranges for therapeutic and preventive effects. 

## Figures and Tables

**Table 1:** The results of curcumin supplementation in PCOS.

Characteristics of Study and Dose of Curcumin	Impact on Parameters in PCOS	Results (Curcumin-Placebo)	*p*	References
Meta-analysis of three random-ized-control trials (168 patients) 500–1500 mg per day 6–12 weeks	Fasting glucose	−2.77	0.001	[25,26,27,28]
	Fasting insulin	−1.33	0.002	
	HOMA-IR	−0.32	0.002	
	QUICKI	0.010	0.005	
	TC	−12.45	0.011	
	HDL	−1.92	0.018	
	LDL	−6.02	0.567	
	TG	8.22	0.639	

PCOS—polycystic ovary syndrome; HOMA-IR—homeostasis model assessment measuring insulin resistance; QUICKI—quantitative insulin sensitivity check index; TC—total cholesterol, HDL—high-density lipoprotein; LDL—low density lipoprotein; TG—triglyceride.

**Table 2:** The results of supplementation nano-curcumin on metabolic parameters in metabolic syndrome and diabetes mellitus.

Characteristics of the Study and Dose of Nano-Curcumin	Impact on Parameters in Metabolic Syndrome	Results	*p*	References
		(Nano-Curcumin-Placebo)		
Randomized double-blind clinical trial (43 patients); 80 mg per day of nanocur-cumin; 3 months	Fasting glucose	−6.9 ± 55.2	0.642	[37]
	Fasting insulin	−1.1 ± 5.6	0.253	
	HbA1c	−0.05 ± 1.59	0.124	
	HOMA-IR	0.3 ± 1.9	0.309	
	HOMA-ß	−5.7 ± 48.2	0.882	
	TC	−5.1 ± 37.4	0.356	
	HDL	3.2 ± 13.07	0.142	
	LDL	1.6 ± 42.9	0.218	
	TG	−60.5 ± 121.7	0.024	
Randomized clinical trial (70 type 2 diabetic patients); 80 mg per day of nano-curcumin (SinaCur-cumin®); 3 months	Fasting glucose	−17.12 ± 40.38	0.657	[39]
	HbA1c	−0.91 ± 1.11	0.013	
	TC	−15.45 ± 44.75	0.889	
	HDL-C	1.83 ± 13.11	0.952	
	LDL-C	−16.41 ± 30.93	0.046	
	TG	−6.7 ± 67.52	0.772	
	BMI	−1.34 ± 1.88	0.001	
(30 patients); 80 mg per day nano-curcumin; 2 months	Fasting glucose		0.016	[35]
	Fasting insulin	-	0.017	
	HOMA-IR	-	0.006	
	TC	190.06 ± 32.89	0.786	
	HDL-C	40.52 ± 9.08	0.15	
	LDL-C	108.23 ± 20.46	0.459	
	TG	176.30 ± 11.34	0.32	
	BMI	32.07 ± 4.15	0.816	
	Afamin	-	0.047	

BMI—body mass index; HbA1c—glycated hemoglobin A1c; HOMA-ß—homeostasis model assessment of ß-cell dysfunction; HOMA-IR—homeostasis model assessment measuring insulin resistance; HOMA-TC—total cholesterol; HDL—high-density lipoprotein; LDL—low-density lipoprotein; TG—triglyceride.

**Table 3:** The results of curcumin supplementation in nine randomized trials on metabolic parameters in NAFLD.

Characteristics of the Study and Dose of Curcumin	Impact on Metabolic Parameters in NAFLD	Results	*p*	References
		(Curcumin-Placebo)		
Meta-analysis of nine ran-domized controlled trials; 500–1500 mg per day 2–3 months	Fasting glucose	0.221	0.027	[41]
	Fasting insulin	−0.487	0.003	
	HbA1c	−0.159	0.213	
	HOMA-IR	0.365	0.031	
	TC	0.645	0.002	
	HDL	0.88	0.139	
	LDL	−1.028	0.028	
	TG	0.608	0.065	
	ALT	−0.458	0.049	
	AST	−0.784	0.032	
	BMI	−0.179	0.058	

HbA1c—glycated hemoglobin A1c; HOMA-IR—homeostasis model assessment measuring insulin resistance; TC—total cholesterol; HDL—high-density lipoprotein; LDL—low -density lipoprotein; TG—triglyceride; ALT—alanine transaminase; AST—aspartate transaminase; BMI—body mass index.

**Table 4:** The results of nano-curcumin supplementation in nine randomized controlled trials on metabolic parameters predicting risk for cardiovascular disease.

Characteristics of the Study and Dose of Nano-Curcumin	Impact on Metabolic Parameters	Results	*p*	References
		(Curcumin-Placebo)		
Meta-analysis of nine random-ized control trials GRADE (510 patients); 6–12 weeks 40–120 mg per day	Fasting glucose	221,218.1	0.001	[33]
	Fasting insulin	22,121.21	<0.001	
	HbA1c	22,120.66	0.081	
	HOMA-IR	22,120.28	<0.001	
	QUCIKI	22,123.34	0.554	
	TC overall	22,120.53	0.945	
	TC < 200	221,210.9	<0.001	
	TC ≥ 200	5.77	<0.001	
	HDL overall	2.01	0.028	
	HDL < 40	7.61	<0.001	
	HDL ≥ 40	22,123.59	0.562	
	LDL overall	22,120.14	0.986	
	LDL < 100	221,213.7	<0.001	
	LDL ≥ 100	22,129.76	0.404	
	TG overall	25.53	0.29	
	TG < 150 mg/dL	221,224.9	<0.001	
	TG ≥ 150 mg/dL	22,122.74	0.858	
	BMI: 25–29.9	221,227.2	0.002	
	BMI > 30	221,218.1	0.001	
	CRP	22,121.29	0.003	
	IL-6	22,122.78	<0.0001	

GRADE—Grading of Recommendations Assessment of Development and Evolution Working Groups guidelines. BMI—body mass index; CRP—C-reactive protein; IL-6—interleukin-6; HbA1c—glycated hemoglobin A1c; HOMA-ß—homeostasis model assessment of ß-cell dysfunction; HOMA-IR—homeostasis model assessment measuring insulin resistance; TC—total cholesterol; HDL—high-density lipoprotein; LDL—low density lipoprotein; TG—triglyceride; QUICKI—quantitative insulin sensitivity check index.
